# Fusion of WiFi, Smartphone Sensors and Landmarks Using the Kalman Filter for Indoor Localization

**DOI:** 10.3390/s150100715

**Published:** 2015-01-05

**Authors:** Zhenghua Chen, Han Zou, Hao Jiang, Qingchang Zhu, Yeng Chai Soh, Lihua Xie

**Affiliations:** 1 School of Electrical and Electronics Engineering, Nanyang Technological University, 50 Nanyang Ave, Singapore 639798, Singapore; E-Mails: zouh0005@ntu.edu.sg (H.Z.); jiangh@ntu.edu.sg (H.J.); zhuq0004@ntu.edu.sg (Q.Z.); EYCSOH@ntu.edu.sg (Y.C.S.); ELHXIE@ntu.edu.sg (L.X.); 2 EXQUISITUS, Centre for E-City, School of Electrical and Electronics Engineering, Nanyang Technological University, 50 Nanyang Ave, Singapore 639798, Singapore

**Keywords:** indoor localization, WiFi, PDR, landmarks, Kalman filter

## Abstract

Location-based services (LBS) have attracted a great deal of attention recently. Outdoor localization can be solved by the GPS technique, but how to accurately and efficiently localize pedestrians in indoor environments is still a challenging problem. Recent techniques based on WiFi or pedestrian dead reckoning (PDR) have several limiting problems, such as the variation of WiFi signals and the drift of PDR. An auxiliary tool for indoor localization is landmarks, which can be easily identified based on specific sensor patterns in the environment, and this will be exploited in our proposed approach. In this work, we propose a sensor fusion framework for combining WiFi, PDR and landmarks. Since the whole system is running on a smartphone, which is resource limited, we formulate the sensor fusion problem in a linear perspective, then a Kalman filter is applied instead of a particle filter, which is widely used in the literature. Furthermore, novel techniques to enhance the accuracy of individual approaches are adopted. In the experiments, an Android app is developed for real-time indoor localization and navigation. A comparison has been made between our proposed approach and individual approaches. The results show significant improvement using our proposed framework. Our proposed system can provide an average localization accuracy of 1 m.

## 1. Introduction

The ability to localize an individual person has resulted in numerous applications, including location-based control, personalized advertisement, evacuation, *etc*. However, the Global Positioning System (GPS) cannot provide location-based services (LBS) with sufficient localization accuracy in indoor environments, due to signal shielding, although it is an optimal choice for outdoor environments. Therefore, developing an accurate and reliable indoor localization system is an emergent task.

One of the most popular techniques in indoor localization is based on the received signal strength (RSS) of WiFi. Due to the variation of WiFi signals, a WiFi-based localization system tends to have large fluctuations. The most commonly-applied technique in RSS-based localization is the fingerprinting approach, which requires manual collection of a huge dataset for training [1,2]. Moreover, the fingerprinting approach requires a re-training process when the environment is altered. In order to overcome these problems, we propose a weighted path loss (WPL) algorithm, which has already been successfully applied in RFID-based localization in [3,4]. First, the distance between a router and a smartphone is calculated using the log-distance path loss model [5]. Then, a weight is determined based on the reciprocal of the distance. Finally, the location of a smartphone is determined by the summation of the weighted locations of routers.

Another widely-adopted localization technique is pedestrian dead reckoning (PDR), which determines the current position based on the previous position, step length and walking direction of the pedestrian [6]. Many parameters, including the initial point, step detection, step length and walking direction, will affect the localization accuracy of the PDR approach [7]. In a short range, the PDR approach can provide very high localization accuracy. However, it will drift along with the walking distance, because only the relative information is leveraged in this approach. Recent research tends to fuse WiFi-based and PDR techniques together to achieve better localization accuracy [5,8–13].

Human activity recognition [14,15] using smartphones may provide a new opportunity for indoor localization. Activities, like turning, going upstairs or downstairs, taking elevators, taking escalators and passing through doors, can be treated as landmarks [16]. The location of turns, stairs, elevators, escalators and doors can be determined when the map information is available. When these activities are detected based on smartphone sensors, we will obtain the location of the pedestrian. Landmarks can be treated as new initial points of a PDR algorithm, which is very sensitive to the estimation error of the initial points. Moreover, they can eliminate system cumulative errors periodically, which cannot be removed using fusion algorithms.

Even though a lot of work has been done in this area, some critical issues still need to be explored and resolved to enhance the accuracy and effectiveness. Recent approaches tend to implement the system in a server instead of a smartphone, which is resource limited. However, in a real situation, localization and navigation services require the system to provide real-time information in smartphones. Therefore, how to develop a lightweight sensor fusion algorithm that can be implemented in a smartphone is a crucial task. In addition, many unresolved problems in WiFi- and PDR-based localization techniques need to be tackled. In this work, we propose to fuse these two techniques together with landmarks using a Kalman filter. Previous works [8,11–13] mainly focused on employing a particle filter, which is time consuming and unsuitable for real-time implementation. In this work, we formulate the problem in a linear perspective. Then, a Kalman filter can be leveraged to solve this fusion problem effectively. For WiFi localization, we intend to apply the WPL algorithm, which has been shown to be accurate and effective. In the PDR approach, some important parameters need to be specified, including the initial point, step length and walking direction. The details will be introduced in subsequent sections.

The rest of the paper is organized as follows: Section 2 introduces some related works in the literature. Section 3 presents WiFi WPL, PDR and identification of landmarks, as well as the fusion technique. Section 4 contains the experimental setup and the discussion of the experimental results. Section 5 concludes the paper and provides some potential future work.

## 2. Related Works

Sensor fusion for localization starts with fusing GPS and inertial sensors in outdoor environments [17–19]. In normal situations, GPS can provide the exact location in real time. However, in the case that GPS is blocked at certain intervals or we want to save energy by lowering the duty cycle of GPS, then sensor fusion is necessary.

In indoor environments, since GPS is not available, other techniques, such as WiFi [20], ultrasound [21] or ultra-wideband (UWB) [22], can be applied to obtain an estimated location instead of GPS. Among them, WiFi is the most popular one, because WiFi routers are widely available and deployed in most buildings. Recent sensor fusion work for localization in indoor environments tends to fuse WiFi and PDR approaches.

The first work considering the integration of WiFi and PDR for indoor localization is found in [8]. The key idea is that these two independent techniques can correct each other by using a particle filter and Kalman filter algorithms. The particle filter is mainly used for smoothing the WiFi fingerprinting approach, while PDR is applied to provide the movement function of particles. In the PDR method, one of the most important parameters is the pedestrian walking direction, which is derived from the integration of gyroscope data. This integration will drift along with the walking distance. Therefore, a Kalman filter is employed to compensate for the drift using the trajectory in the WiFi fingerprinting method. Moreover, in order to achieve 3D localization, barometers are employed to identify climbing or going down stairs. A box containing inertial sensors was attached to the belt of the pedestrian during the experiment. The final achieved accuracy of the system was 1.53 m in the experiments.

Another work proposed in [11] focused on fusing inertial sensor, indoor map and WLAN RSS together to navigate the pedestrian in indoor environments. The gyroscope data are applied to obtain the walking direction, and the 3D acceleration is leveraged to detect each step. They also explored the relationship between step length and step frequency. Then, a linear model is fitted for step length estimation. In WLAN RSS-based localization, the fingerprinting technique is used. In order to reduce the computational load of the whole system, they proposed an obstacle line grouping method for the indoor map. In the simulation, they showed great improvements after fusion of all of the information.

Similar work can be found in [12]. They proposed fusing a low-cost acceleration, WLAN signals and map information to achieve pedestrian tracking. Similar to the other works, they applied the fingerprinting technique for WLAN-based localization, which is treated as a benchmark for comparison. For the PDR approach, the walking length is estimated from an empirical equation. They claimed that the performance of the algorithm is not sensitive to the accuracy of walking length estimation. The walking direction is sampled from a uniform distribution. Finally, a particle filter is applied for sensor fusion. In simulations and real experiments, the proposed algorithm achieved great improvements over unfused algorithms.

One of the most recent works can be found in [23], where the authors proposed a localization framework, which combines motion information from smartphone sensors and the WiFi fingerprinting technique using a Bayes filter. They applied the term belief, which represents the conditional probability distribution over all possible states referring to the users' coordinates. In the motion model, the belief was updated based on the pedestrian dead reckoning approach. Then, the WiFi fingerprinting method, which leverages the rank-based scheme and Spearman's footrule distance, instead of the existing nearest neighbor method, was employed for the correction of belief. Experiments were conducted under two contrasting environments. The experimental results showed that their system outperforms other WiFi-only approaches and requires less training and maintenance cost. Moreover, it can even work in a sparse WiFi environment.

In the literature, researches tend to formulate PDR as a nonlinear function. Therefore, particle filters are leveraged to tackle this sensor fusion problem. Due to the computational load of particle filters, this approach can only be suitable for off-line experiments and running on a server. In real situations, a pedestrian needs to obtain real-time localization and navigation. In order to achieve real-time implementation on a resource limited smartphone platform, we need to develop a simple and efficient fusion algorithm. Therefore, we formulate the fusion problem in a linear perspective, then a Kalman filter algorithm, which is computationally light, can be applied. In order to enhance the performance of the system, we attempt to improve the accuracy of the individual subsystems, *i.e.*, WiFi and PDR approaches. In WiFi localization, we apply a WPL approach, which is efficient and simple for implementation. In the PDR approach, several parameters need to be specified, including the initial point and walking direction. Since the initial point is vital in PDR-based localization, we combine landmarks to restart the algorithm and reset the accumulative error of the system. Another important parameter that needs to be specified is the walking direction. Traditional approaches tend to apply the magnetometer or gyroscope independently to estimate the walking direction. However, we found that magnetometers are very sensitive to electronic devices, and gyroscopes will drift in estimation, because of the integration process. Therefore, we propose to fuse these two techniques using another Kalman filter.

## 3. Methodology

Individual localization techniques have their own strengths and weaknesses. For example, the WiFi-based approach has large fluctuations, because of WiFi signal variations. In a short range, the PDR approach can produce high localization accuracy, but it will drift along with the walking distance. The drift in PDR can be partially reset by landmarks. We proposed to combine these two approaches with landmarks using a Kalman filter. The details for each individual subsystem are introduced in the following sections.

### 3.1. Weighted Loss Path of WiFi

The most popular WiFi positioning technique is the fingerprinting approach, which contains two phases, off-line training and on-line testing. In the off-line training phase, a large amount of data, including the RSS and the corresponding location information, is collected. Then, machine learning techniques are applied to obtain the relationship between RSS and location information. In the on-line testing phase, based on the model learned in the training phase, the location information can be derived according to a real-time WiFi RSS value. The WiFi fingerprinting approach requires tedious manual collection of data for training and the re-training of the model when the environment changes.

In order to overcome the drawbacks of the fingerprinting approach, we present a WPL algorithm, which has already been successfully applied in an RFID-based localization system [3,4]. Assume $s_{t}^{i}$is the RSS of the router *i* at time step *t*, then, based on signal propagation model in [5], we obtain:

(1) $$s_{t}^{i}=PL_{0}+10\alpha \log\left(d_{t}^{i}\right)$$

where *PL*_0_ is the reference path loss coefficient, *α* is the path loss exponent and $d_{t}^{i}$is the distance between the router *i* and the device at time step *t*. Based on Equation (1), $d_{t}^{i}$can be expressed as:

(2) $$d_{t}^{i}=10^{\frac{s_{t}^{i}-PL_{0}}{10\alpha }}$$

Suppose we have *N* routers and that the distance between the device and routers can be expressed as a vector $\left\{d_{t}^{1},d_{t}^{2},\cdots ,d_{t}^{N}\right\}$at time step *t*. Then, the weight of each router can be calculated as:

(3) $$w_{t}^{i}=\frac{\frac{1}{d_{t}^{i}}}{\sum_{i=1}^{N}\frac{1}{d_{t}^{i}}}$$

Finally, the location of the device, (*x*, *y*), can be calculated as:

(4) $$\left(x,y\right)=\sum_{i=1}^{N}w_{t}^{i}\left(x_{i},y_{i}\right)$$

where (*x_i_*, *y_i_*) is the location of the router *i*.

Considering that our system is running on a resource-limited smartphone instead of a server, the lightweight WPL algorithm is more suitable than the popular fingerprinting approach, which requires manual collection of a huge dataset for training and normally performs heavy load machine learning techniques in the algorithm. Moreover, when the environment changes, the WPL algorithm only needs to adjust two parameters, *i.e.*, *PL*_0_ and *α*, in Equation (1), while the fingerprinting approach requires manual collection of the data again and a tedious re-training process. Therefore, the WPL algorithm is more suitable for our real-time implementation.

### 3.2. Pedestrian Dead Reckoning

Smartphone inertial sensors, including the accelerometer, magnetometer and gyroscope, can provide unique means for localization. Intuitively, performing double integration of the acceleration will yield the walking distance of a pedestrian. However, due to the inherent noise and vibrations during walking, the double integration of acceleration will diverge quickly [16,24]. One realistic solution is the PDR approach, which determines the next position using the previous position, step length and walking direction, which is expressed as follows:

(5) $${\text{X}}_{t}={\text{X}}_{t-1}+L_{t}\left[\begin{array}{c} \sin\left(\theta_{t}\right)\\ \cos\left(\theta_{t}\right) \end{array}\right]$$

where **X***_t_* is the position at time step *t*, *L_t_* is the step length and *θ_t_* is the walking direction at time step *t*. Several important issues need to be solved, such as initial location estimation, step detection, step length estimation and walking direction estimation.

#### 3.2.1. Initial Location Estimation

The PDR approach only provides relative information. Therefore, the accuracy in estimating the initial position will directly influence the accuracy of the entire PDR localization. Since we do not have any prior information of the initial position, the only location information comes from the WiFi positioning system at the beginning. Due to the importance of the initial position estimation, we leverage the landmarks, whose positions are known, as new starting points to restart the algorithm when the pedestrian reaches these landmarks. In addition, landmarks will be helpful in resetting accumulative errors of the system caused by unknown bias in sensors. The detailed definition and identification of landmarks will be introduced in Section 3.3.

#### 3.2.2. Step Detection

Steps can be detected based on the periodic pattern of vertical acceleration during walking. In real situations, the raw data of acceleration are noisy. Therefore, we perform a smoothing function on the raw data to reduce the noise effect. Assume {*a_t_*,*t* ∈ 1, …,*K*} is the vertical acceleration time series; the *m* order smooth function output at time step *t*, $a_{t}^{m}$, can be calculated as:

(6) $$a_{t}^{m}=\frac{\sum_{i=t}^{t+m-1}a_{i}}{m}$$

**Figure 1.**
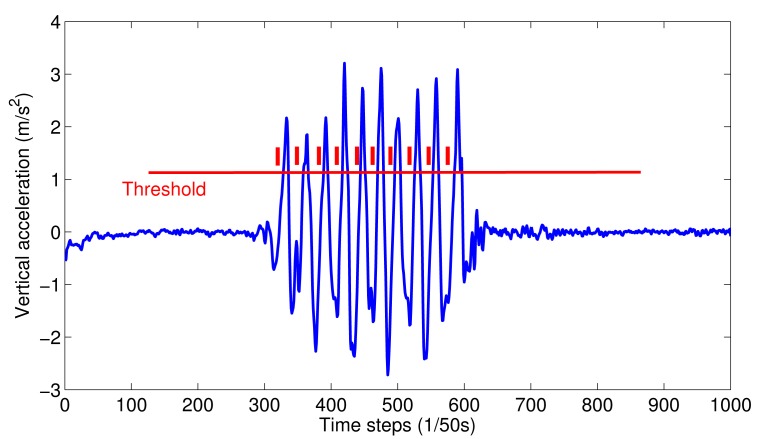
Vertical acceleration pattern after smoothing.

Figure 1 shows the vertical acceleration pattern after smoothing. Then, a simple threshold method can be applied to identify each step easily.

#### 3.2.3. Step Length Estimation

The step length of different pedestrian varies a lot. Even for the same person, the step length changes dramatically during walking. Some advanced models have been proposed in the literature. The simplest method applied a constant step size in different modes, such as walking, jogging and running [7]. Alternatively, the authors in [25] presented a linear relationship between step length and the height of the pedestrian, expressed as:

(7) L=height∗k

where the coefficient, *k*, can be determined by the gender of the pedestrian. These two approaches are relatively simple to implement; however, they neglect the variance of step length during walking. A more sophisticated approach is proposed in [6], where the authors presented a relationship between acceleration magnitude and step length. The expression is as follows:

(8) $$L=\beta {\left(a_{\max}-a_{\min}\right)}^{1/4}$$

where *β* is the coefficient that needs to be adjusted for different subjects. This approach takes the dynamics of step length during walking into consideration. In this work, we adopt this approach for step length estimation.

#### 3.2.4. Walking Direction Estimation

One method for estimating pedestrian walking direction is based on the orientation sensor output, which is a combination of magnetometer and accelerometer readings in smartphones [26]. One of the orientation sensor outputs, *i.e.*, the azimuth reading, is the angle between the smartphone pointing direction and geographical north. Since this angle is determined by the magnetometer output, which will be affected by electronic devices, we intend to compensate for this effect by the gyroscope output, which provides the angular acceleration without any effect of electronic devices. Suppose that the angle, the angular velocity and the angular acceleration are *Q_t_*, *V_t_* and *u_t_* at time step *t*, respectively. We define the system state as **S***_t_* = [*Q_t_ V_t_*]*^T^*, and the input of the system is defined as *u_t_*. Then, based on Newton's law, we can formulate the system as:

(9) $${\text{S}}_{t}={\text{AS}}_{t-1}+\text{B}u_{t}+\text{w}$$

where $\text{A}=\left[\begin{array}{cc} 1 & dt\\ 0 & 1 \end{array}\right]$and $\text{B}=\left[\begin{array}{c} dt^{2}/2\\ dt \end{array}\right]$are coefficients, and **w** denotes the Gaussian noise of the system with zero mean and variance *ϕ*. The observation of the system comes from one of the orientation sensor outputs, the azimuth reading, which is defined as *O_t_*. Then, the observation function can be expressed as:

(10) $$O_{t}={\text{CS}}_{t}+r$$

where **C** = [1 0] and *r* denotes the Gaussian noise of the magnetometer output with zero mean and variance *φ*

The Kalman filter [27] is applied to solve this linear problem with the assumption of Gaussian noises. The expression contains two parts:

**Predicting:**

(11) $${\text{S}}_{t|t-1}={\text{AS}}_{t-1|t-1}+\text{B}u_{t}$$

(12) $${\text{P}}_{t|t-1}={\text{AP}}_{t-1|t-1}{\text{A}}^{T}+\phi$$

**Updating:**

(13) $${\text{K}}_{t}={\text{P}}_{t|t-1}{\text{C}}^{T}{\left({\text{CP}}_{t|t-1}{\text{C}}^{T}+\varphi \right)}^{-1}$$

(14) $${\text{S}}_{t|t}={\text{S}}_{t|t-1}+{\text{K}}_{t}\left(O_{t}-{\text{CS}}_{t|t-1}\right)$$

(15) $${\text{P}}_{t|t}=\left(\text{I}-{\text{K}}_{t}\text{C}\right){\text{P}}_{t|t-1}$$

### 3.3. Identification of Landmarks

Landmarks whose positions are known have specific sensor patterns, which make them identifiable in the environment. The motivation of introducing landmarks is that the accuracy of the PDR approach highly depends on the accuracy of initial location estimation. When the initial location estimation is very poor, the error will accumulate in terms of the initial error in the subsequent steps. Landmarks can provide accurate new starting points for the PDR algorithm when the pedestrian reaches these landmarks [16]. In addition, they are also helpful in resetting the cumulative error of the whole system.

The landmarks investigated in this work include turns, elevators, escalators, stairs and doors. The sensors involved in the identification of these landmarks are the accelerometer, magnetometer, gyroscope, barometer and WiFi.

**Figure 2.**
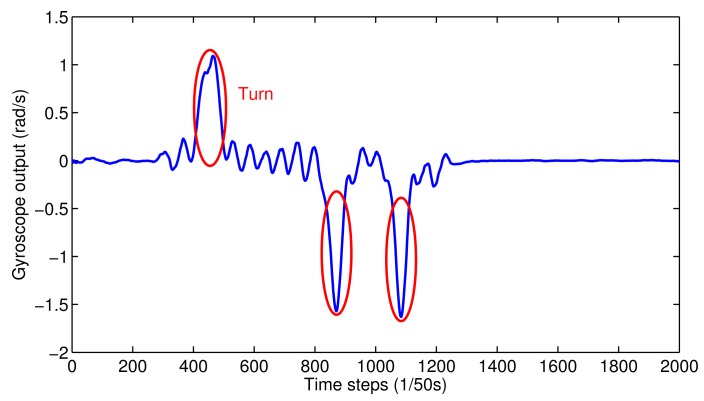
Identification of turns.

Turns: Due to the topology constraints in indoor environments, turns can be identified using angular or direction-related sensors. The most direct way to recognize turns is to use the magnetometer reading. However, the magnetometer output will be affected by electronic devices, which makes it unstable for the recognition of turns. An alternative way to do this is by leveraging the gyroscope information, which measures angular acceleration without the influence of any equipment. After smoothing the gyroscope output, turns can be easily distinguished, which is shown in Figure 2. In addition, based on the direction of the pulse in the figure, left or right turns can be separated. In real situations, many turns can be detected. If the distance of two turns is smaller than localization accuracy, we may make a wrong decision, which will lead to a bias in the system. In order to avoid this problem, we only consider turns that are unique in the path as landmarks.

Elevators: Based on the unique pattern of vertical acceleration data, taking elevators can be easily identified. There will be a hyper-gravity and a hypo-gravity process, shown as a positive impulse and a negative impulse of vertical acceleration, respectively [16]. Moreover, the length between the impulses reflects how many floors the pedestrian goes up or down, which can be important for multi-floor localization.

Escalators: The acceleration pattern of taking escalators is similar to being stationary. In order to distinguish them, the magnetometer data are used. In the stationary condition, the variance of magnetometer data is small. However, since the motors in the escalators will dramatically influence the magnetic field, the variance of the magnetometer reading will be large when taking escalators.

**Figure 3.**
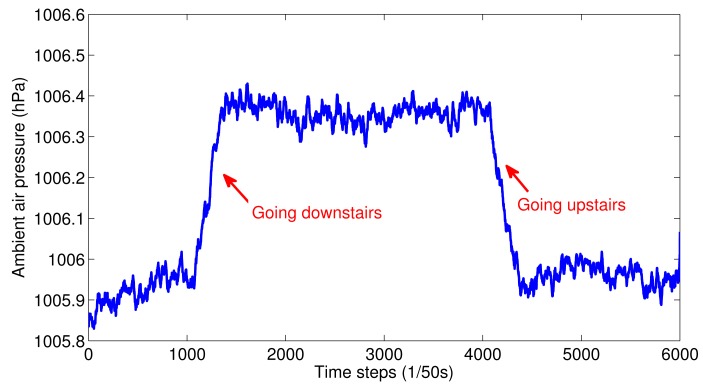
Ambient air pressure for going upstairs and downstairs.

Stairs: Going upstairs or downstairs has a similar pattern of acceleration as normal walking. An effective way to distinguish these two activities is to use the barometers in smartphones [28]. It is known to all that the higher the elevation, the lower the ambient air pressure. Therefore, there will be a decrease in ambient air pressure when going upstairs and an increase in ambient air pressure when going downstairs. Figure 3 shows an example of ambient air pressure change for going upstairs and downstairs.

**Figure 4.**
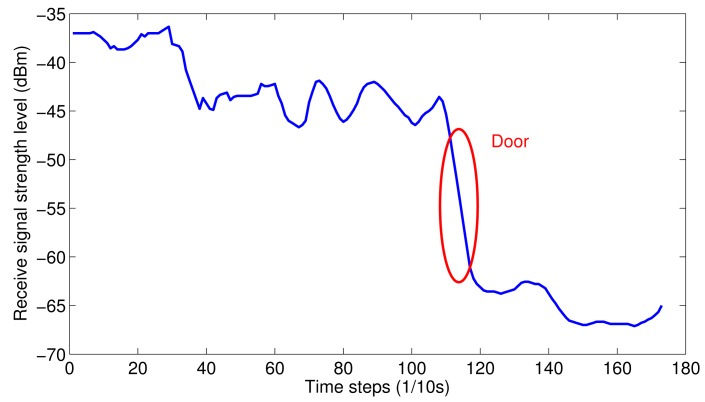
Received signal strength of WiFi when passing through a door.

Doors: The landmarks of doors contain two phases of acceleration: a low value for opening the door and a periodic pattern for walking out. If only based on this phenomenon, many false positive events will be produced. Another prominent property of passing through a door is the big change of the received signal strength of WiFi, as shown in Figure 4. Based on this property, doors can be easily identified.

### 3.4. Sensor Fusion with the Kalman Filter

WiFi WPL and PDR approaches have unique properties in localization. Among them, the WiFi WPL approach can provide the exact location. However, it is not robust enough due to the variation of the WiFi signal. The PDR approach can generate the relative location; it is very accurate in a short range, but it will drift along with the walking distance. These two independent approaches can be combined to compensate for the weaknesses of each other. The WiFi WPL method will be helpful in correcting the drift of the PDR approach, while the PDR approach will smooth the variation in the WiFi WPL method.

The potential sensor fusion techniques for WiFi and PDR approaches include the particle filter [29], hidden Markov model [30], Kalman filter [27], *etc*. In real situations, users need to know their locations in real time. Therefore, the whole system should be running on smartphones instead of servers. Because of this constraint, light computation algorithms will be preferred, because of limited resources in smartphones. In terms of problem formulation, we tackle the problem in a linear perspective, then a Kalman filter, which is computationally light, can be applied instead of a particle filter, which is widely used in the literature.

Assume **X***_t_* is the 2D coordinate of the pedestrian and **d***_t_* =*L_t_* [sin*θ_t_* cos*θ_t_*]*^T^* is the input where *L_t_* is the step length and *θ_t_* is the walking direction at time step *t*. Then, based on the PDR approach, the state transition function of the sensor fusion framework is as shown below:

(16) $${\text{X}}_{t}={\text{FX}}_{t-1}+{\text{Gd}}_{t}+\text{v}$$

where **F, G** are identity matrices and **v** denotes the Gaussian noise of the motion model with zero mean and covariance matrix **M** [31]. The observation function can be obtained based on the WiFi WPL output, ${\text{Z}}_{t}=\sum_{i=1}^{N}w_{t}^{i}\left(x_{i},y_{i}\right)$, where $w_{t}^{i}$is the weight of router *i* at time step *t* and (*x_i_*, *y_i_*) refers to the location of router *i*. The observation function can be expressed as follows:

(17) $${\text{Z}}_{t}={\text{HX}}_{t}+\text{p}$$

where **p** denotes the Gaussian noise of WiFi WPL output with zero mean and covariance matrix **N** [32]. Since it is a direct observation problem, **H** is an identity matrix.

The Kalman filter [27] is applied for this sensor fusion. The algorithm contains two processes, predicting and updating.

**Predicting:**

(18) $${\text{X}}_{t|t-1}={\text{FX}}_{t-1|t-1}+{\text{Gd}}_{t}$$

(19) $${\text{P}}_{t|t-1}={\text{FP}}_{t-1|t-1}{\text{F}}^{T}+\text{M}$$

**Updating:**

(20) $${\text{K}}_{t}={\text{P}}_{t|t-1}{\text{H}}^{T}{\left({\text{HP}}_{t|t-1}{\text{H}}^{T}+\text{N}\right)}^{-1}$$

(21) $${\text{X}}_{t|t-1}={\text{X}}_{t|t-1}+{\text{K}}_{t}\left({\text{Z}}_{t}-{\text{HX}}_{t|t-1}\right)$$

(22) $${\text{P}}_{t|t}=\left(\text{I}-{\text{K}}_{t}\text{H}\right){\text{P}}_{t|t-1}$$

## 4. Evaluation

In order to evaluate the performance of the proposed model, two real experiments have been conducted. The comparisons have been made between the proposed fusion approach and the individual approaches of WiFi WPL and PDR with landmarks.

### 4.1. Experiment Setup

**Figure 5.**
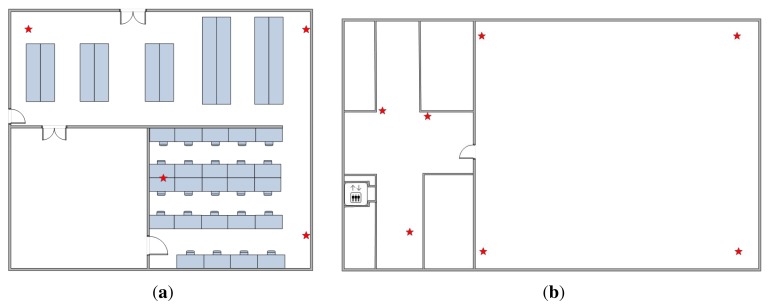
Layout. (**a**) The research lab; (**b**) the testbed.

**Figure 6.**
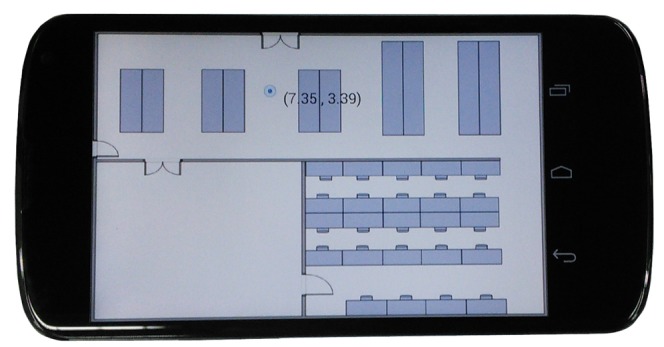
The user interface.

The two experiments are performed in a typical research lab and a designed testbed at the NTU (Nanyang Technological University) campus. The sizes of the lab and the testbed are 19.0 m × 16.3 m and 27.5 m × 16.4 m, respectively. Figure 5 shows the layout of the research lab and the testbed. The red stars are the locations of the APs (access points). The device involved in the experiments is a Google Nexus 4 smartphone running the Android 4.4 operation system. We developed an Android app for the experiments. One user interface is shown in Figure 6. The circle shows the position of the user, and the number represents the corresponding coordinate. We define the top left corner as the coordinate origin; the x-axis points to the right, and the y-axis points downwards. Note that we only consider the hand-held situation; the other situations, such as putting the phone in a pocket or a bag, are our future works. In order to evaluate the performance of the model, we compare our proposed sensor fusion model with WiFi WPL and PDR with landmarks approaches. To obtain the ground truth of the trajectory, we mark the ground with a 1-m grid and apply a camera to record the whole walking process; then, we manually measure the location of each step of the pedestrian.

### 4.2. Experimental Results and Discussions

**Figure 7.**
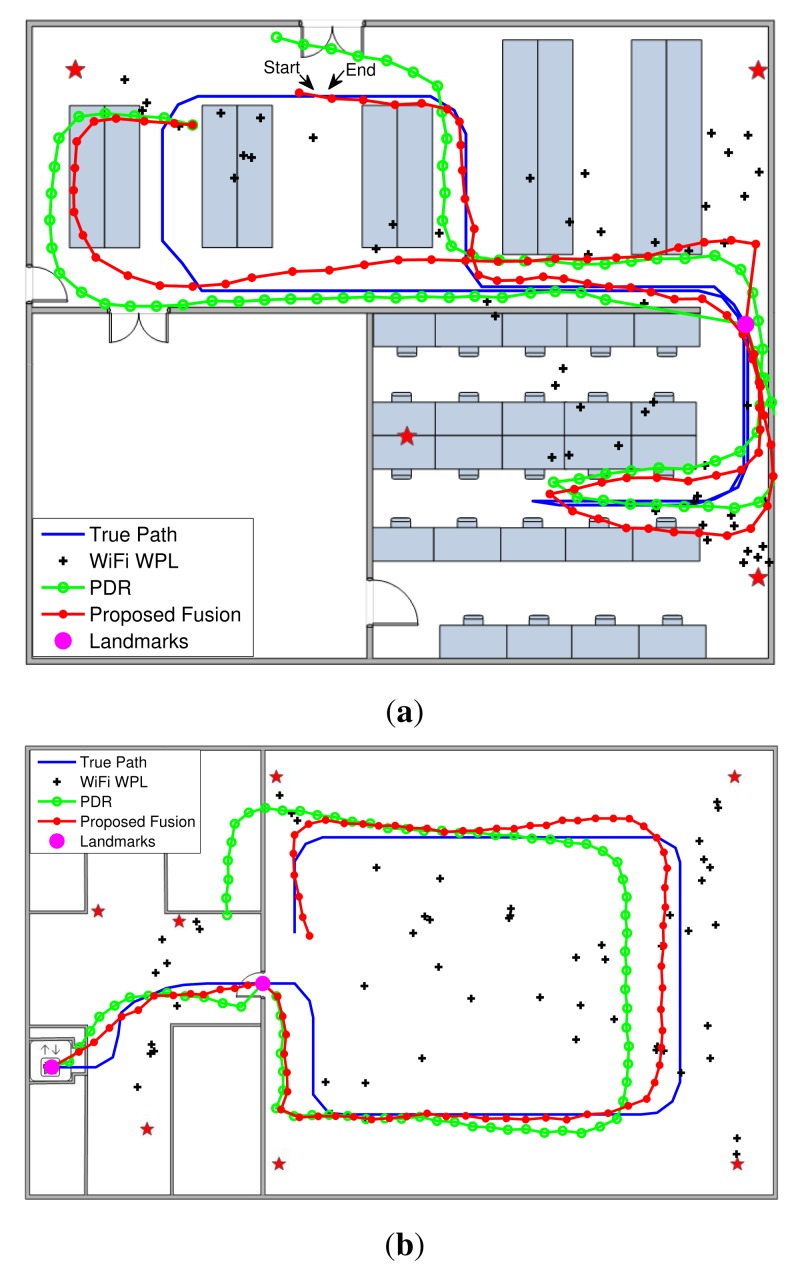
The trajectories of the true path, the WiFi weighted path loss (WPL) model, the pedestrian dead reckoning (PDR) with landmarks and the proposed fusion model for the two experiment setups. (**a**) The research lab; (**b**) the testbed.

The first experiment was conducted in the research lab. Due to the physical constraints caused by the facilities, a lot of physical turns are involved in the experiment. Based on the criterion introduced in Section 3.3, only one turn, shown as a magenta circle in Figure 7a, satisfies the set criterion. According to the true trajectory in Figure 7a, only one landmark of a turn is applied in the experiment. One additional experiment has been performed in the designed testbed. Since the testbed is relatively empty, no physical turns are involved in the experiment. Therefore, no landmarks of the turns can be used. According to the true trajectory in Figure 7b, two landmarks, an elevator and a door, are leveraged in the experiment.

**Figure 8.**
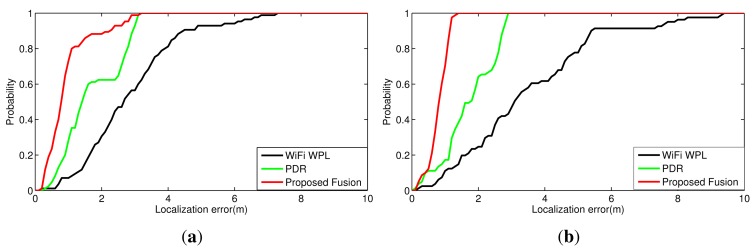
Cumulative distribution functions of the localization error for the three approaches. (**a**) The research lab; (**b**) the testbed.

Figure 7 shows the trajectories of the true path, WiFi WPL model, PDR with landmarks and our proposed fusion model for the two experimental setups. The corresponding cumulative distribution functions (cdfs) of localization errors for the three approaches are demonstrated in Figure 8. In the first experiment, the mean localization errors of WiFi WPL and PDR with landmarks are 2.8977 m and 1.7547 m, respectively, while that of the proposed fusion model with the Kalman filter is 0.9945 m. After sensor fusion of the WiFi, PDR and landmarks, we reduce the localization errors by 65.7% and 43.3%, respectively, when compared to the WiFi WPL and PDR with landmarks approaches. Since only one landmark is employed in the middle of the path in the first experiment, the localization error is relatively large before arriving at the landmark. After restarting from the landmark, PDR provides a high localization accuracy within a short range and then slowly drifts, while the proposed fusion algorithm corrects the drift using the WiFi WPL output. Clearly, the proposed sensor fusion approach takes the advantages of the individual techniques, including PDR, WiFi WPL and landmarks, to achieve high localization accuracy. In the second experiment, the mean localization errors of WiFi WPL and PDR with landmarks are 3.5189 m and 1.7727 m, respectively, while that of the proposed fusion model with the Kalman filter is 0.8492 m. After sensor fusion of WiFi, PDR and landmarks, we reduce the localization errors by 75.9% and 52.1%, respectively, when compared to the WiFi WPL and PDR with landmarks approaches. Note that we did not adjust the parameters of WiFi WPL in the new environment. Therefore, the localization accuracy of WiFi WPL is slightly poorer than that in the first experiment. However, the entailed fusion system can still achieve a high localization accuracy, *i.e.*, 0.8492 m, when combining with PDR and landmarks. The tracking starts from an elevator, which is a known landmark. Before arriving at the second landmark, which is a door, PDR has relatively small localization error, but the walking direction has some deviations at the beginning, which may be caused by the magnetic effect of the motors in the elevator. However, the proposed fusion algorithm reduces the deviations by the correction of WiFi WPL. After passing through the door, PDR has high localization accuracy in a short range and then slowly drifts. However, our proposed algorithm follows the true path well when fusing PDR and WiFi WPL. In summary, the proposed system has smaller variation than WiFi WPL and a lower drift than PDR, and it provides an average localization accuracy of 1 m.

## 5. Conclusions and Future Works

In this work, we have proposed a sensor fusion of WiFi, pedestrian dead reckoning and landmarks by using a Kalman filter algorithm. In the WiFi-based localization, we apply a weighted pass loss algorithm, which is accurate and simple to implement. In order to reduce the effect of the initial point estimation error in the PDR approach, we employ landmarks that have specific patterns of smartphone sensor data. Note that we have assumed that the locations of landmarks are known. Another important issue in the PDR approach is the estimation of the walking direction. Since magnetometers in smartphones are easily affected by electronic devices, we combine gyroscope data with magnetometer output using another Kalman filter to obtain a more robust estimation of the walking direction. In real situations, users need to obtain their real-time location information by smartphones. Since smartphones are resource-limited platforms, we formulate the problem in a linear perspective in the sensor fusion part. This is so that a Kalman filter algorithm, which is computationally light, can be applied to solve this problem instead of a particle filter, which is widely used in the literature. Finally, we develop an Android app for real experiments in a research lab and a designed testbed. In order to demonstrate the improvement of this sensor fusion approach, a comparison has been made between our proposed approach and the individual approaches of WiFi WPL and PDR with landmarks. The experiment results show an impressive improvement in localization accuracy. Our proposed approach achieves a mean localization accuracy of 1 m.

In summary, the main contributions of this work are:

- We proposed a sensor fusion framework for combining WiFi WPL, PDR and landmarks for indoor localization using a Kalman filter and demonstrated an average localization accuracy of 1 m.
- In the WiFi localization, we used the weighted path loss algorithm, which is simple and effective, so that our system can run on a resource-limited smartphone platform.
- In the PDR approach, landmarks are introduced to tackle the initial estimation error of the PDR-based approach, and to improve the accuracy of walking direction estimation, a Kalman filter is employed to fuse magnetometer and gyroscope readings.

In future works, we will mainly focus on multi-floor localization, where few works have been done. As we mentioned in Section 3.3, landmarks, such as stairs, escalators and elevators, can be easily identified. Based on this information, we can infer the floor where the pedestrian is located. Combined with our proposed model, we can achieve multi-floor localization. Note that, when WiFi signals are not available in certain regions, the PDR with landmarks approach can still provide coarse localization information.
